# Supplementary material for: Glass-to-Glass Fusion Bonding Quality and Strength Evaluation with Time, Applied Force, and Heat
Source: Micromachines (Basel). 2022 Nov 2;13(11):1892. doi: 10.3390/mi13111892 (PMC9695810; doi:10.3390/mi13111892)
Supplement: Supplementary file 1 [file micromachines-13-01892-s001.zip › micromachines-1988577-supplementary.pdf]

Supplemental Information for:

Glass-to-glass fusion bonding quality and strength evaluation with time, applied force, and heat

Nhi N. Trinh[1], Leslie A. Simms[2], Bradley S. Chew[2], Alexander S. Weinstein[2], Valeria La Saponara[2], Mitchell M. McCartney[1,2,3], Nicholas J. Kenyon[2,3,4], Cristina E. Davis[1,2,3,*]

[1]Biomedical Engineering, University of California Davis, Davis, CA

[2]Mechanical and Aerospace Engineering, University of California Davis, Davis, CA

[3]UC Davis Lung Center, University of California Davis, Davis, CA

[4]VA Northern California Health Care System, Mather, CA

[5]Department of Internal Medicine, University of California Davis, Davis, CA

[*]Corresponding author: cedavis@ucdavis.edu

Table S1 *– Wilcoxon rank sum tests were conducted for bonding percentage and flexural strength with and without surface activation.*

**Independent Variable**	**Wilcoxon rank sum test *P*-values**
	With SAB – Without SAB
Bonding Percentage	1.0000
Flexural Strength	1.000

Table S2 *– Wilcoxon rank sum tests were conducted for bonding percentage and flexural strength between 1 h-2 h, 2 h-4 h, and 1 h-4 h values.*

**Independent Variable**	**Wilcoxon rank sum test *P*-values**		
	1 h-2 h	2 h-4 h	1 h-4 h
Bonding Percentage	0.7000	0.2000	0.7000
Flexural Strength	0.4000	0.4000	1.0000

Table S3*- Wilcoxon rank sum tests were conducted for bonding percentage and flexural strength between 550 N-840 N, 840 N-1110 N, and 550 N-1110 N values.*

**Independent Variable**	**Wilcoxon rank sum test *P*-values**		
	550 N-840 N	840 N-1110 N	550 N-1110 N
Bonding Percentage	0.7000	0.2000	0.7000
Flexural Strength	0.7000	0.4000	0.7000

***Table S4**- Wilcoxon rank sum tests were conducted for bonding percentage and flexural strength between 550 °C-650 °C, 650 °C-700 °C, and 550 °C-700 °C values.*

**Independent Variable**	**Wilcoxon rank sum test *P*-values**		
	550 °C-650 °C	650 °C-700 °C	550 °C-700 °C
Bonding Percentage	0.1000	0.1000	0.2000
Flexural Strength	1.000	0.4000	0.2000

Surface Roughness Measurements

Further testing was conducted on the external surface roughness after the bonding procedure to evaluate the finish of the glass wafers. The applications of this bonding process are attractive for application that requires additional deposition of material on the external surface. The surface profile of the wafers is done using a DekTak XT (Bruker, Billerica, MA, USA), where the root mean square average (RMS) of the profile height deviations from the mean line can be calculated with Equation 2:

$$RMS = [\frac{{Z_1}^2 + {Z_2}^2 + \cdots + {Z_N}^2}{N}]^{1/2} \quad (1)$$

where Z is the height of all the peaks and valleys, and N is the number of peaks. The surface profile was measured at 5 general areas on the wafer in two directions: perpendicular and parallel to the primary flat. This gave a total of $n = 10$ data points per wafer sample, where all the RMS values were calculated for each data point and averaged for each individual wafer.

Table S5 – Calculated RMS and Wilcoxon rank sum tests were conducted on all samples made for fusion bonding

**Bonding Parameter**	**Testing sample**	**Calculated RMS (µm)**	**Wilcoxon rank sum test P-values**
Surface Activation Bonding	WOSAB	0.23	1.00 (WOSAB – WSAB)
	WSAB	0.24	
Hold Time	1 h	0.14	0.40 (1 h – 2 h)
	2 h	0.24	0.40 (2 h – 4 h)
	4 h	0.28	1.00 (1 h – 4 h)
Applied Pressure	550 N	0.16	0.70 (550 N – 840 N)
	840 N	0.78	0.40 (840 N – 1110 N)
	1110 N	0.66	0.40 (550 N – 1110 N)
High Temperature	550 °C	0.01	1.83e-4 (550 °C – 650 °C)
	650 °C	0.16	1.83e-4 (650 °C – 750 °C)
	750 °C	1.42	1.83e-4 (550 °C – 750 °C)
